# Supplementary material for: Interaction Effect between Physical Activity and the BDNF Val66Met Polymorphism on Depression in Women from the PISMA-ep Study
Source: Int J Environ Res Public Health. 2022 Feb 12;19(4):2068. doi: 10.3390/ijerph19042068 (PMC8872527; doi:10.3390/ijerph19042068)
Supplement: Supplementary file 1 [file ijerph-19-02068-s001.zip › ijerph-1561381-supplementary.pdf]

Supplementary

Table S1. Relationships between Val66Met genotype and physical activity variables. Statistically significant results are highlighted in bold.

	Total sample (3123)			Women (1554)			Men (1569)		
	Val/Val (1947)	*Met allele carriers (1176)*	*Adjusted* OR (95% CI), P*	*Val/Val (969)*	*Met allele carriers (585)*	*Adjusted** OR (95% CI), P*	*Val/Val (978)*	*Met allele carriers (591)*	*Adjusted** OR (95% CI), P*
Physical activity									
None	829 (43%)	431 (37%)		431 (44%)	245(42%)		398 (41%)	186 (31%)	
Yes	1118 (57%)	745 (63%)	**1.26 (1.08–1.47), 0.002**	538 (56%)	340 (58%)	1.09 (0.88-1.35), 0.403	580 (59%)	405 (69%)	**1.48 (1.19–1.84), $5.28x10^{-4}$**
Intensity of physical activity									
None	829 (43%)	431 (37%)		431 (44%)	245(42%)		398 (41%)	186 (31%)	
Light	385 (20%)	239 (20%)	1.04 (0.87–1.25), 0.65	216 (22%)	134 (23%)	1.04 (0.81-1.33), 0.761	169 (17%)	105 (18%)	1.05 (0.80–1.38), 0.716
Moderate	589 (30%)	399 (34%)	1.17 (1.00–1.36), 0.055	285 (30%)	167 (28%)	0.94 (0.74-1.82), 0.608	304 (31%)	232 (39%)	1.41 (1.14–1.76), $1.74x10^{-3}$
Vigorous	144 (7%)	107 (9%)	1.21 (0.92–1.58), 0.164	37 (4%)	39 (7%)	1.75 (1.09-2.80), 0.019	107 (11%)	68 (12%)	1.01 (0.72–1.40), 0.957

Abbreviations: CI, confidence interval; OR, odds ratio; *P*, p-value. * Adjusted by age, BMI and sex. ** Adjusted by age and BMI. Statistically significant results are highlighted in bold
